# Effect of Gambogic Acid on miR-199a-3p Expression and Cell Biological Behavior in Colorectal Cancer Cells

**DOI:** 10.1155/2021/5140621

**Published:** 2021-08-26

**Authors:** Xiaodong Wang, Yingchun Li, Haihua Zhou, Ning Han, Linlin Pan, Chen Yu

**Affiliations:** Department of Anus & Intestine Surgery, Taizhou People's Hospital, Taizhou Clinical Medical College of Nanjing Medical University, Taizhou 225300, Jiangsu, China

## Abstract

Colorectal cancer (CC), as a malignancy threatening life and health, has a rising incidence in recent years. It has been reported that gambogic acid (GA) has antitumor activity in various tumors, but its effect on CC remains to be elucidated. In this investigation, the influence of GA nanoparticles on microRNA-199a-3p (miR-199a-3p) in CC was analyzed to provide a reliable reference for future clinical practice. Through PCR detection, we first determined that miR-199a-3p presented low expression in CC and had a significant effect in predicting the onset and prognosis of CC. Through in vitro experiments, the enhanced CC cell viability after inhibition was determined; however, decreased cell viability and increased miR-199a-3p level were also observed after GA nanoparticles addition. Hence, GA nanoparticles may influence CC cell biological behaviors by modulating miR-199a-3p, providing a novel treatment scheme for CC in the future.

## 1. Introduction

As a malignant disease threatening life and health, colorectal cancer (CC) has a very high prevalence on the global scale [1, 2]. According to research reports, the incidence of CC is as high as 4-5%, and the susceptibility is linked to personal characteristics and habits, such as age, history of chronic diseases, and lifestyle [3, 4]. In such a case, the gut microbiome plays a related role, while malnutrition can trigger CC through a chronic inflammatory mechanism [5]. The prevalence of CC rises along with the development of society and the change of people's living habits [6], and it is more pervasive in middle-aged people over 40 years old, with more males than females [7]. At the beginning of CC, the cure rate is extremely high. Whereas, the disease can be ignored easily due to the absence of obvious symptoms in the early stage, and as a result, most patients are in the middle stage when diagnosed [8, 9]. Referring to previous relevant data, it is found that the prognosis of patients in the advanced stage is unfavorable after surgical treatment, which seriously affects the therapeutic effect [10]. In recent years, as the traditional Chinese medicine (TCM) has risen to prominence in clinical treatment, its treatment of major diseases such as malignancies has attracted increasing attention [11].

Gambogic acid (GA) is a potential anticancer compound [12], which is extracted from *Garcia hanburyi*'s resin and has potent anticancer activity [13]. Studies have found that GA has an obvious inhibitory effect on liver cancer [14]. Besides, many related studies confirmed that GA has certain curative effects on breast cancer, lymphoma, skin cancer, and gastric cancer [15]. Moreover, miR-199a-3p interferes with the onset and progression of various malignant tumors. Author Callegari E [16] observed that in hepatocellular carcinoma transgenic mouse models, MTOR and PAK4 pathways were modulated and tumor growth was suppressed by miR-199a-3p. Author Cui Y [17] revealed enhanced cisplatin sensitivity of ovarian cancer cells by miR-199a-3p. Whereas, the lack of research data hinders the elucidation of the clinical value of miR-199a-3p in CC. Considering its clinical manifestations in other malignant tumors, we presumed that it might benefit the treatment of CC.

In recent years, the research of nanomedicine is a research hotspot in the medical field. Nanomedicine can not only improve the effect and half-life of the drug but also reduce its toxic and side effects [18]. GA nanobodies have also been confirmed to have a higher clinical application value than traditional GA [19]. Therefore, this study will analyze the effects of GA nanobodies on the expression of miR-199a-3p in colorectal cancer cells as well as cell proliferation and apoptosis and provide new ideas and theoretical foundations for the treatment of colorectal cancer in the future.

## 2. Materials and Methods

### 2.1. Patient Data

From April 2013 to May 2015, fifty-six CC patients treated at Taizhou People's Hospital, Taizhou, Jiangsu, PR China, were selected as the research group (RG), while 61 healthy individuals as the control group (CG). The study was approved by the Ethics Committee of the Taizhou People's Hospital, Jiangsu, PR China, and all patients provided written informed consent.

### 2.2. Inclusion and Exclusion Criteria

All the included patients were confirmed by endoscopy, laboratory examination, and imaging and met the diagnostic criteria for CC. All the primary lesions were CC, and all the patients were aged 35 or older.

Patients with pregnancy or lactation were excluded, as well as those with other tumors or immunodeficiency.

### 2.3. Cell Data

Human CC cell line SW480 and normal human colon epithelial cell line NCM-460 were purchased from BeNa Culture Collection, a subsidiary of ATCC, USA, while gambogic acid was from Shanghai PureOne Biotechnology. After resuscitation, the cells were inoculated into the corresponding culture medium and cultivated in a 5% CO_2_ incubator at 37°C. The passage was carried out when the confluence reached 80%, and cells used in experiments were in a growth period.

### 2.4. Cell Intervention

After transfection with the corresponding vector according to the Lipofectamine 2000 kit instructions, the miR-199a-3p-NC group (intervened by miR-199a-3p negative control), miR-199a-3p-mim group (intervened by miR-199a-3p mimic sequence), and miR-199a-3p-inh group (intervened by miR-199a-3p inhibitor sequence) were set up.

### 2.5. Preparation of GA Nanoparticles

Dephosphatidylcholine (PC) 60 mg, cholesterol (Chol) 30 mg, and GA 4 mg was mixed and add 15 mL of chloroform to dissolve in a rotary evaporator (0.01 MPa, 30°C) to form a film, remove the solvent, and then increase the vacuum to remove traces of chloroform. Add phosphate-buffered saline solution and glass beads (2 mm), spin, and hydrate for 5 min. After 10 minutes of sonication, GA nanoparticles were prepared.

### 2.6. GA Nanobody Intervention Cell

SW480 was added to the culture medium containing GA nanoparticles to incubate for 48 hours and then subcultured. When the growth length reached 80%, the follow-up test was performed.

### 2.7. PCR

The total RNA, after extraction, was reverse transcribed into cDNA according to PrimeScript^TM^ RT reagent Kit and used SYBR Green PCR Master Mix for PCR detection. The internal reference was U6, and the expression level was calculated by 2^−△△CT^.

### 2.8. CCK-8

Cells seeded in the 96-well plates following 24, 48, 72, and 96 h of culture were immersed in 20 *μ*L CCK-8, and the absorbance (450 nm) of each well was measured following another 4 h of culture. After the removal of culture solution, 200 *μ*L GA was added to each well, followed by the replenishing of culture solution, with a final concentration of 4.0 *μ*g/mL. After the addition of 20 *μ*L CCK-8, the absorbance (450 nm) of each well was determined following another 4 h of culture, in order to draw the cell growth curve.

### 2.9. Flow Cytometry Test

The apoptosis level of the cells was observed by flow cytometry assay. The cells were diluted at 2 × 10^3^ cells/mL by ice Annexin V-FITC binding buffer. Cells were cultured in fresh Gibco 1640 solution with 0 *μ*g/mL as a blank group. They were treated with 10 *µ*L of propidium iodide (PI 20 *µ*g/ml) and 5 *µ*L of Annexin V-FITC/PI (10 *µ*g/ml) cell apoptosis assay 24 later and cultivated at ambient temperature for 10 min for apoptosis determination. Finally, the apoptosis level of the cells was instantly observed by a flow cytometry equipment (BD Biosciences, State of New Jersey, USA).

### 2.10. Transwell Experiment

Transwell upper chamber and lower chamber were added with cells adjusted to 1 × 10^6^/mL and fetal bovine serum culture medium, respectively. After 24 h, the cells in the upper chamber were wiped off, immobilized, stained, and counted under the microscope.

### 2.11. Western Blot

Fishing extraction from RIPA lysate, the total protein was processed for SDS-PAGE electrophoresis isolation and membrane transfer. Then, it was processed for 2 h of sealing with 50 g/L skimmed milk powder and 24 h of cultivation mixed with Bax, Bcl-2 (1 : 1000, AB_2533042), and *β*-actin (1 : 1000, AB_11004139) at 4°C, followed by TBST rinsing and 2 h of cultivation with II antibody (1 : 2000). Image *J* analyzed the band gray value. All antibodies were purchased from ThermoFisher (Massachusetts, USA).

### 2.12. Statistical Analysis

Data were processed by SPSS22.0 and visualized using Graphpad7. The average value of the experimental results was calculated and recorded as mean ± standard deviation; the comparison between groups was realized by the independent sample *t*-test, the comparison among groups by one-way ANOVA and LSD post-hoc test. Receiver operating characteristic (ROC) curves analyzed the predicted value. Statistical difference was considered when *P* < 0.05.

## 3. Results

### 3.1. miR-199a-3p Expression Profiles in CC

**Figure 1:**
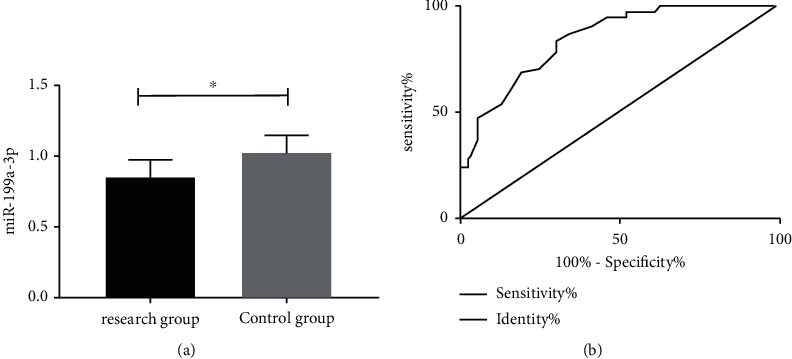
miR-199a-3p expression. (a) miR-199a-3p expression in the research group and control group. (b) Predictive significance of miR-199a-3p in the onset of CC. Note: ^∗^*P* < 0.05.

miR-199a-3p showed lower levels in RG (0.83 ± 0.13) than in CG (1.02 ± 0.12) (*P* < 0.05, Figure 1(a)). ROC revealed that miR-199a-3p < 0.925, and the sensitivity, specificity, area under the curve (AUC), and standard error were 83.61%, 69.64%, 0.846, and 0.035, respectively (*P* < 0.001, Figure 1(b), Table 1).

### 3.2. Influence of miR-199a-3p on Patient Prognosis

**Figure 2:**
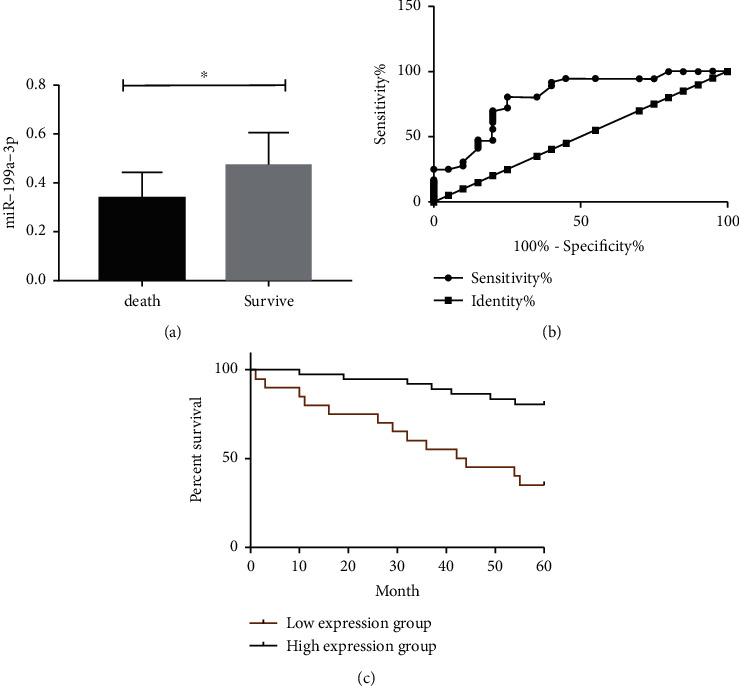
Influence of miR-199a-3p on patient prognosis. (a) miR-199a-3p expression in patients with survival and death. (b) ROC curves of miR-199a-3p for predicting patient death. (c) Prognostic survival curves. Note: ^∗^*P* < 0.05.

The 5-year follow-up rate was 100%. The follow-up results indicated that miR-199a-3p was (0.34 ± 0.09) among the 20 dead, while miR-199a-3p was (0.50 ± 0.11) among the 36 survivors (Figure 2(a) and Table 2). ROC exhibited that when miR-199a-3p < 0.35, it had a sensitivity of 80.56%, a specificity of 65.00%, an AUC of 0.804, and standard error of 0.064 (*P* < 0.001, Figure 2(b) and Table 2). The patients were subdivided into high and low expression groups based on the cutoff value, and prognosis and survival curves determined a remarkably higher mortality rate in the low expression group compared with the high expression group (*P* < 0.05, Figure 2(c) and Table 2).

### 3.3. Influence of miR-199a-3p on SW480 of Human CC Cell Strain

**Figure 3:**
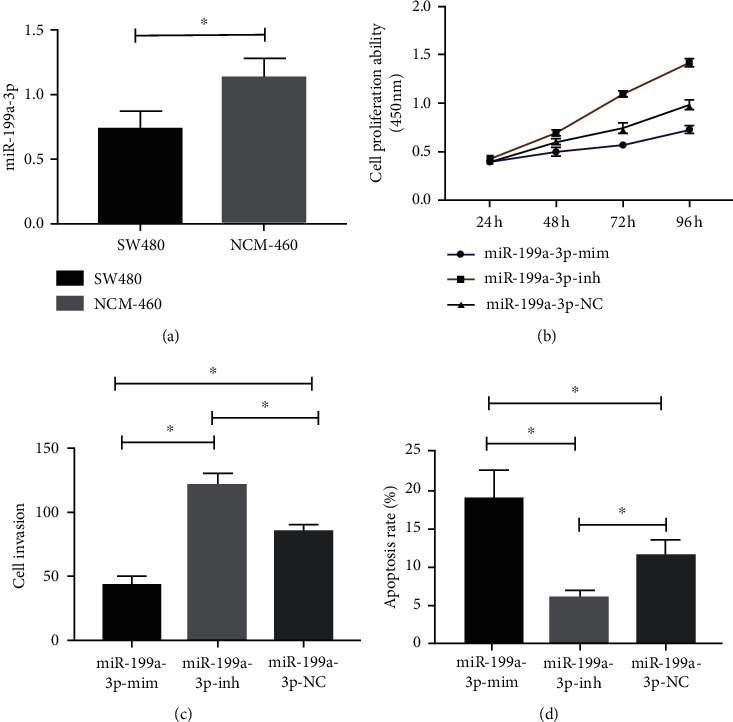
Influence of miR-199a-3p on SW480. (a) miR-199a-3p expression. (b) Cell proliferation. (c) Cell invasion. (d) Apoptosis rate. Note: ^∗^*P* < 0.05.

miR-199a-3p expression in human CC cell strain SW480 and normal human colon epithelial cell NCM-460 was detected. The results identified statistically lower miR-199a-3p in SW480 than in NCM-460 (*P* < 0.05, Figure 3(a)). Besides, the miR-199a-3p-mim group showed declined proliferation and invasion capacity and elevated apoptosis rate, while the miR-199a-3p-inh group presented enhanced proliferation and invasion ability and reduced apoptosis (*P* < 0.05, Figures 3(b)–3(d)).

### 3.4. Influence of GA on Human CC Cell Strain SW480

**Figure 4:**
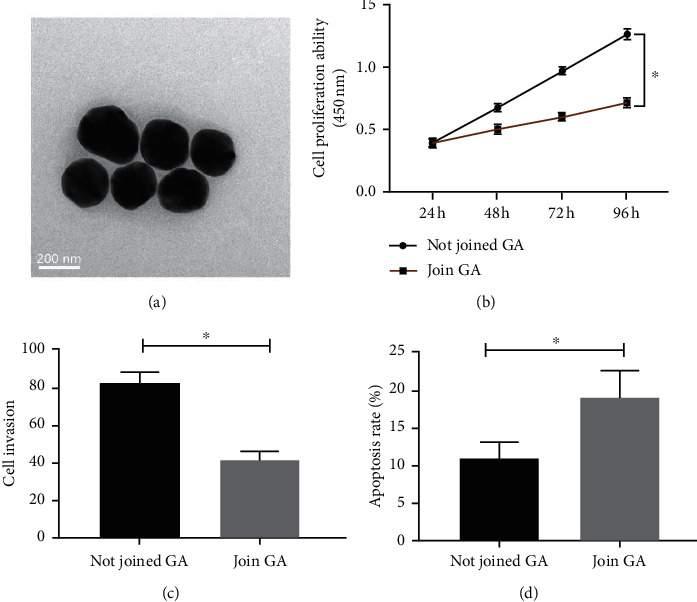
Influence of GA on SW480. (a) Observe GA nanoparticles under TEM. (b) Cell proliferation. (c) Cell invasion. (d) Apoptosis rate. Note: ^∗^*P* < 0.05.

Under TEM, the particle size of GA nanobodies is about 200 nm (Figure 4(a)). Changes in proliferation, invasion, and apoptosis rate of human CC cell strain SW480 after GA intervention were analyzed. It showed that the tumorigenic phenotype of CC cells SW480 was reduced following GA intervention, and the apoptosis rate was increased compared with those without GA intervention (*P* < 0.05, Figures 4(b)–4(d)).

### 3.5. miR-199a-3p Expression Changes in Cells following GA Addition

**Figure 5:**
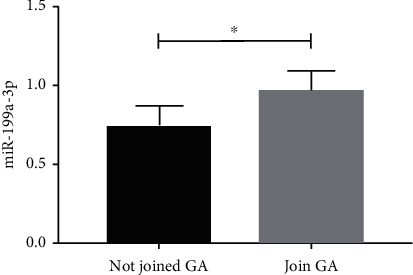
miR-199a-3p expression changes in cells following GA addition.

miR-199a-3p expression in the CC cells after GA intervention (0.96 ± 0.13) was significantly higher than the CC cells without GA intervention (0.73 ± 0.14) (*P* < 0.05, Figure 5).

### 3.6. Influence of miR-199a-3p on Human CC Cell Strain SW480 following GA Addition

**Figure 6:**
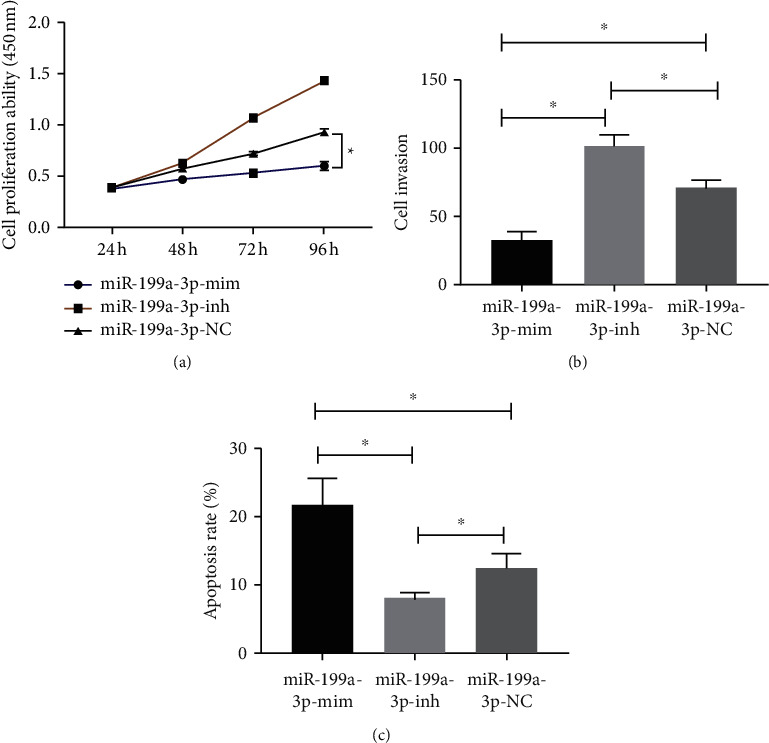
Influence of miR-199a-3p on SW480 following GA addition. (a) Cell proliferation. (b) Cell invasion. (c) Apoptosis. Note: ^∗^*P* < 0.05.

Transfection of GA-interfered SW480 resulted in the lowered proliferation and invasion ability and increased apoptosis in miR-199a-3p-mim group, while the reverse results were obtained in the miR-199a-3p-inh group (*P* < 0.05, Figure 6).

## 4. Discussion

Due to people's unhealthy living habits, the incidence of CC is on the rise [20, 21]. Molecular exploration and analysis is now a trending topic in the research of malignant tumor diseases, which carries huge implications for future prevention and treatment of tumors [22]. This study, through preliminary investigation of the influence of miR-199a-3p and GA nanoparticles on CC, may provide potential basis for future clinical practice.

First, low miR-199a-3p in serum was observed in CC patients, implying that miR-199a-3p may interfere with CC progression. Similar results were obtained in the previous research [23], which can testify to our experimental results. Then, for the sake of determining its clinical significance, we determined miR-199a-3p's predictive value in CC by ROC curve analysis. The results showed that the predictive sensitivity and specificity of miR-199a-3p for CC were 83.61% and 69.64%, respectively, demonstrating its plausible predictive effect on the onset of CC, for it not only has a better response ability to CC but also has a superior recognition specificity. The current tumor markers commonly used in the clinic, such as CEA, CA125, and CA199, can basically show a strong response to all tumor diseases [24]. There are also pieces of evidence pointing out that the traditional cancer markers increase in conditions such as chronic inflammatory diseases or tissue injuries [25], so the ability of early screening for tumor diseases is getting lower and lower. The diagnostic value of miR-199a-3p for CC can effectively make up for the deficiencies of traditional cancer targets, bolster the early diagnosis rate of CC, and improve the prognosis of patients. Furthermore, according to a previous study, miR-199a-3p is a candidate diagnostic marker for renal cell carcinoma [26], which further confirms its significant clinical application potential in the future. Of course, ROC curve analysis usually requires a large number of research bases for analysis, so that the cutoff value obtained and the diagnostic performance can be more accurate [27]. However, the included case number is relatively small, and consequently, there may be errors in reverifying the results. Therefore, large sample size and multicenter evaluation will provide more potential basis regarding this concept. Subsequently, we analyzed the influence of miR-199a-3p on patient prognosis through the prognostic follow-up. The results identified notably lowermiR-199a-3p levels among the dead, which was basically consistent with the results of our above analysis. And through ROC curve analysis, we found that miR-199a-3p has an excellent predictive value for the prognostic death of CC patients. Moreover, the prognostic survival curves identified an increased risk of death confronted by patients with low miR-199a-3p expression. Based on the above results, we can see that the lower the miR-199a-3p expression is, the more severe the development of CC may be, which also provides a reliable reference for future clinical understanding of the patient's condition.

Although some studies have analyzed the action mechanism of miR-199a-3p [28], the mechanism of its action on CC is not clear. Hence, the biological effects of miR-199a-3p on CC cells were investigated through in vitro experiments. The results revealed enhanced CC cell viability following miR-199a-3p suppression, but increased miR-199a-3p lead to the opposite. Thus, it is clear that low-expressed miR-199a-3p promotes the activity of tumor cells and accelerates CC progression, which is consistent with the previous research [29] and our above experimental. Then, we tested the biological behavior of CC cells under GA intervention and found obviously inhibited cell viability and enhanced apoptosis rate. Hence, GA nanoparticles can play a role in the treatment of CC, which verifies the effect of GA nanoparticles. Furthermore, an increased miR-199a-3p increased was observed under GA nanoparticles intervention, so we speculated that the influence of GA nanoparticles on CC cells might be associated with miR-199a-3p. Finally, miR-199a-3p mimics and inhibitors were introduced into CC cells under GA nanoparticles intervention. The results were found to be roughly the same as those after miR-199a-3p transfection as mentioned above, except that cell viability and apoptosis showed more distinct differences under GA nanoparticles intervention. The study has indicated that GA could effectively inhibit the progression of the nonsmall cell lung cancer via inducing the autophagy progression of the tumor cells [30]. Therefore, we can preliminarily infer that through modulating miR-199a-3p, GA nanoparticles exert their effect on CC cells. However, due to the short experimental period, how miR-199a-3p interferes with the long-term prognosis of CC patients remains unresolved. Besides, the influence of GA nanoparticles on CC cells may not only be carried out by affecting miR-199a-3p, so further exploration of other mechanisms is warranted. Moreover, we did not compare the effect of cisplatin and other commonly used CC treatment drugs in clinical practice with that of GA nanoparticles, so it is still unclear which drug has a more significant effect on tumor cells. Last but not least, the deeper and more intricate relationship and relevant pathways of GA nanoparticles and miR-199a-3p on CC cells are worthy of further experimental analysis.

## 5. Conclusion

GA may affect CC cell biological behaviors by modulating miR-199a-3p, which usually shows low expression in CC, providing a novel treatment approach for CC in the future.

## Figures and Tables

**Table 1:** Primer sequence.

	Forward primer	Reverse primer
miR-199a-3p	5′-GCCACAGTAGTCTGCACAT-3′	5′-CAGTGCGTGTCGTGGAGT-3′
U6	5′-CTCGCTTCGGCAGCACA-3′	5′-AACGCTTCACGAATTTGCGT-3′

**Table 2:** Diagnostic effect of miR-199a-3p on CC.

	miR-199a-3p
AUC	0.846
Standard error	0.035
95% CI	0.777–0.914
Cutoff	>0.925
Sensitivity (%)	83.61
Specificity (%)	69.64
Youden index	53.25
*P*	<0.001

## Data Availability

The data used to support the findings of this study are available from the corresponding author upon request.
